# A study of the effects of different group involvement on children’s sport behavioral choices: a group preference-based perspective

**DOI:** 10.3389/fpsyg.2025.1632707

**Published:** 2025-10-13

**Authors:** Xiaoyan Fan, Hui Xiao, Tongnian Yang, Qi Liu

**Affiliations:** ^1^School of Physical Education, Nanchang Institute of Technology, Nanchang, Jiangxi, China; ^2^School of Physical Education and Health, Jiangxi Science and Technology Normal University, Nanchang, Jiangxi, China; ^3^School of Athletic Performance, Shanghai University of Sport, Shanghai, China

**Keywords:** sports behavior choice, group involvement, in-group favoritism, parental attachment, peer attachment

## Abstract

**Introduction:**

In order to investigate the characteristics of children’s sport behavioral choices in different group-involvement contexts and the role of group favoritism in this relationship.

**Methods:**

280 children aged 3–8 years were recruited for a psycho-experimental study in two different experimental contexts.

**Results and discussion:**

The results found that (1) in the in-group and out-group involvement situations, the rate of physical activity selection was significantly higher in the in-group than in the out-group, and there was a significant difference, indicating that there was an in-group preference in the selection of physical activities by children aged 3 to 8 years old. (2) There was a significant age difference in the low favorite physical activity choice in the parent-intimate peer involvement context. Negative rank was lower than positive rank in physical activity choice among 3- to 4- and 5- to 6-years-olds, suggesting the presence of parental attachment behaviors among 3- to 4- and 5- to 6-years-olds. In summary, it can be seen that children aged 3–8 years show a clear phenomenon of in-group favoritism in their choice of physical activity, which may be related to children’s early social cognitive development and attachment behavior.

## Introduction

With the introduction of the Double Reduction Policy, China is focusing on the healthy physical and mental growth of students, and physical activity is an essential and important condition for promoting the healthy physical and mental growth of children. How to engage children in more active and positive physical activity became the focus of this study. Social identity theory places individuals at the center of identifying with groups and identifying with their groups through social categorization, resulting in in-groups and out-groups (Tajfel and Turner, 1979). Individuals who treat in-groups in interpersonal interactions show more positive attitudes, make positive evaluations, and develop favorable behavioral and affective attitudes, i.e., in-group favoritism (Zhang et al., 2009). Much of the research on in-group favoritism has been in the areas of task choice or outcome choice (Fenerty and Tiger, 2010), choice of values (Schmidt et al., 2009), choice of sharing behaviors (Chernyak and Kushnir, 2013), belief preferences (Everett et al., 2015), and fair norm enforcement (Zhu et al., 2023). It was found that children between the ages of 5 and 8 years showed in-group favoritism in both first-party contexts and third-party contexts for distributional behavior (Liu et al., 2019). All of the above studies have verified that the phenomenon of in-group favoritism affects children’s psychological development and the development of behavioral choices.

The theory of in-group favoritism suggests that individuals tend to choose activities that are relevant to the group to which they belong when making behavioral choices, i.e., they tend to engage in activities that are favored by the in-group (Kruglanski et al., 2006). This preference is based on the individual’s quest for group identity and social belonging (Volz et al., 2009). Thus, the involvement of different groups in children’s sport behavior choices can have an impact on their sport behavior. Involvement of different groups can shape preferences for particular sports activities (Estabrooks et al., 2012). For example, in physical education classes at school, certain children may be more involved in ball games, while others may prefer other physical activities such as jumping rope or swimming, and such preferences are largely influenced by the group to which they belong, i.e., physical activities favored by the in-group are more likely to appeal to and attract members of that group. In addition, the involvement of different groups can affect the social identity of children’s sports behavior (Vignoles and Moncaster, 2007). The theory of in-group favoritism emphasizes the individual’s sense of identification with the group and social belonging, which is closely related to the degree of social group involvement. When choosing sports activities, children consider the social groups to which they belong, such as classes, grades, clubs, etc., and the level of participation and attitudes of these groups have an impact on their choice of sports behaviors, making them more inclined to choose sports activities that enhance their social identity and thus better connect them to the social groups to which they belong (Steele et al., 2002; Anderson-Butcher et al., 2003). Different group involvement affects children’s attitudes and motivation toward sports behavior (Viciana et al., 2007). It can be seen that when more people in the group to which children belong participate in a particular physical activity, they tend to develop more positive attitudes and higher motivation to participate in that activity, due to the children’s desire to participate in the activity with their peers, to experience social interaction and a sense of belonging, and to receive affirmation and recognition from the group.

Most of the previous studies that have been done on children’s sport behavior choices have been in the areas of age, gender, and influences on sport behavior choices. Children make choices between children of different genders and types of children, with boys choosing boys interested in sports and girls choosing girls interested in resources and emotional leadership (Braza et al., 2012), i.e., children gradually begin to have the ability to make choices between sports based on gender, further suggesting that children’s in-group favoritism has an important role in the relationship between resources and emotions and sport behavior in relation to their peers is significant. Meanwhile, studies have found that participation in sports in early childhood enhances social skills, self-regulation, and pro-social behavior (Harlow et al., 2020). Participation behaviors in physical activity are inconsistent across age levels, with preschoolers focusing on fun games with friends while elementary school-aged children prefer semi-structured and structured activities (Fitzgerald et al., 2009), meaning that friends and group conditions are very important to the process of participation in physical activity for preschoolers. Participation in physical activity and this process of friends and group conditions are very important. Intergroup favoritism, on the other hand, arises within or between groups and shows a tendency to show a clear preference for a particular group, both evaluatively and behaviorally (Chen and Cui, 2015). This leads to the conclusion that the use of good in-group favoritism during physical activity can be effective in increasing children’s initiative to participate in physical activity.

Every choice interprets an importance, and the same should be true for children’s sports behavior choices. Rationalization of sport behavior choices is a priority for children’s physical activity. However, children’s participation in physical activity may be constrained by gender. There are studies that prove that children as young as 2 years old are already aware of the concept of having gender roles (Boyle et al., 2003). Most of the previous research has been on the essential connotations, characteristics, and motivations of sports behaviors, and very few studies have examined the mechanisms of children’s behavioral choices in sports, and there is an even greater lack of research on behavioral choices during children’s kindergarten developmental period. Therefore, this study focuses on the factors influencing children’s sport behavior choices based on group favoritism and its practical implications.

Three main hypotheses are proposed based on the currently available literature:

Hypothesis 1: There is a “group preference” in the choice of sports behaviors among children, and sports activities are also influenced by this phenomenon.

Hypothesis 2: Children’s in-group preference phenomenon will decrease and stabilize with age, and sports behavior choice will be more reasonable. To explore the mechanism of children’s sports behavior choice influence so as to effectively promote the motivation of children’s sports activity participation, stimulate children’s sports activity participation, and enhance children’s sports activity enthusiasm.

Hypothesis 3: Children will show parental attachment and peer attachment in parent-intimate peer involvement, which is related to children’s psychological developmental history.

## Experiment 1

### Materials and methods

#### Participants

Sample size was calculated by *a priori* analysis based on G-Power 3.1.9 software, setting the effect size *f* = 0.25, significance level α = 0.05, and statistical efficacy 1-β = 0.8, and it was found that at least 158 subjects were needed. A total of 300 children were recruited in two general kindergartens and one elementary school in Nanchang City using random cluster sampling, and 20 invalid data were excluded due to missing data and other reasons. Finally, 280 subjects were included, including 82 subjects in the 3–4-years-old group (46 boys, mean monthly age M = 49.22, SD = 6.268); 84 subjects in the 5–6-years-old group (48 boys, mean monthly age *M* = 73.10, SD = 5.28); and 114 subjects in the 7–8-years-old group (58 boys, mean monthly age M = 96.09, SD = 6.12). A 3 (age: 3–4, 5–6, 7–8) × 2 (gender: boys, girls) × 2 (group condition: in-group, out-group) mixed experimental design was used. Considering children’s gender and age as two independent variables, we discussed the characteristics of children’s choice behavior based on group preference by comparing subjects’ sport behavior choices in the in-group-out-group involvement approach and applied the phenomenon of group preference to increase children’s participation and motivation in sport behavior. The study was approved by the Ethics Committee of Jiangxi Normal University of Science and Technology. Informed consent for the experimental content and survey of this study was obtained from the children themselves, the teachers of the children’s school, and their parents in the pre-experimental period.

#### Materials

The main experiment was conducted in a quiet room using a one-to-one format with children alone. The experimental procedure was carried out in three stages; first, the experimenter asked the children to identify the members of the in-group and out-group by presenting pictures of the children in different classes; immediately after that, the children were differentiated according to the degree of their enjoyment of the sports, and the sports with a high degree of enjoyment, those with a medium degree of enjoyment, and those with a low degree of enjoyment were selected. Finally, children complete a selection of items with different levels of favoritism among different groups.

#### Procedure

**FIGURE 1:**
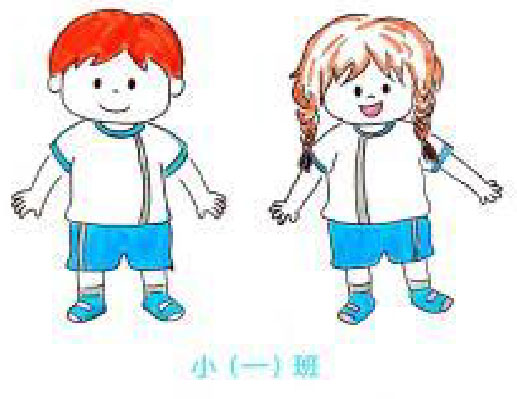
Class1-A.

**FIGURE 2:**
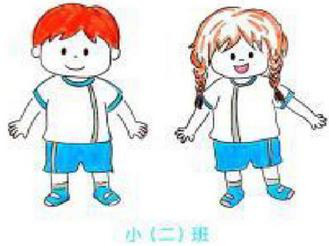
Class1-B.

**FIGURE 3:**
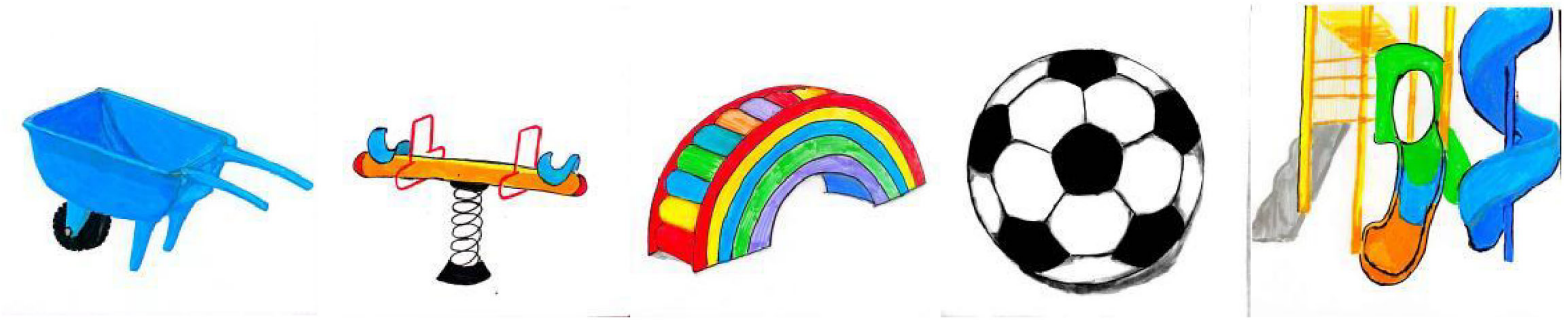
Map of sports programs.

1. Distinguish between in-groups and out-groups. Subjects were presented with two pictures containing class iconicity: a picture of the name logo of the subject’s class and a picture of the name logo of another class. Place a clipart picture of a child with the name of his or her class in front of the subject and tell the subject, “This is a child from your class, and he corresponds to the child in Figure 1.”Place a clipart picture of a child with the name of the class in another class in front of the subject and tell him or her, “This is the child from the next class; he corresponds to the child in Figure 2, but you have never played with him.” During the experiment, the color of the clothes of the clipart photo and the position of the class name sign were counterbalanced between the subjects, and the subjects were made to know that these two children were consistent with their gender and age. The primary subject took out the two pictures and “asked the children if the child in Picture 1 is in your class.” The lead-in words for Picture 2 were consistent with Picture 1 (subjects correctly answered “It’s a child in the class” for in-group relations, and vice versa for out-group relations).

2. Distinguish between sport favorites. Choose 5 pictures of sports items (pushing a unicycle, riding a spring-loaded teeter-totter, walking an arched rainbow bridge, playing soccer, and playing on a slide) that are similar in size, different in color, and different in motion from the items of choice. In the test, the primary test subject presents the subject with a chart of 5 sports and “asks the subject: you have 5 different sports charts in front of you; which is your favorite sport?” The master test recalled the first favorite selected by the subject. The subject is then asked: of the remaining 4, which is your favorite? And so on until the last favorite sport is reached. Then, the subject picked out the charts of the sports that the subjects had chosen as their favorite (high level of favorite), average favorite (medium level of favorite), and least favorite (low level of dislike).

3. Sport behavioral choices under different group involvement conditions. On the basis of the subjects’ distinction between inside and outside groups and sport favoritism, the subjects were tested on their choice of children’s sports behavior in different group-involvement conditions in both inside and outside groups and sport favoritism. In the test, the main test subject presented 3 different favorite sports and pictures of children in the class and children in the class next door to the subject. “Ask the subject: right now, the children in your class are playing your favorite (high degree of liking) sport, would you go along and participate?” “Ask the subject: the children in your class are playing your average favorite (medium level of liking) sport; would you go along and participate?” “Ask the subject: if the children in your class are playing a sport that you like least (low degree of liking), would you go along and participate?” Test the children in the class next door, asking the same words as above. Both groups needed to be asked about three separate sports with different levels of liking (see Figure 3).

### Data processing

The data were analyzed using SPSS 26.0 software, and descriptive statistics were used to analyze the mean age of the children in months. Given the ordinal nature of sports preferences (ordered data) and the violation of normality/sphericity assumptions in the within-subjects design used in this study, we employed Wilcoxon signed-rank tests (for paired comparisons) and chi-square tests (for frequency differences). Wilcoxon tests compared in-group vs. out-group and parent vs. peer conditions by ranking within-subject differences (e.g., higher positive ranks for in-group participation). Chi-square tests quantified proportional differences (e.g., participation rates across age/gender). The above non-parametric methods can robustly handle ordered data without assuming interval scaling or normal distribution.

## Results

### The effect of in-group-out-group involvement on behavioral choices in sport programs

**FIGURE 4:**
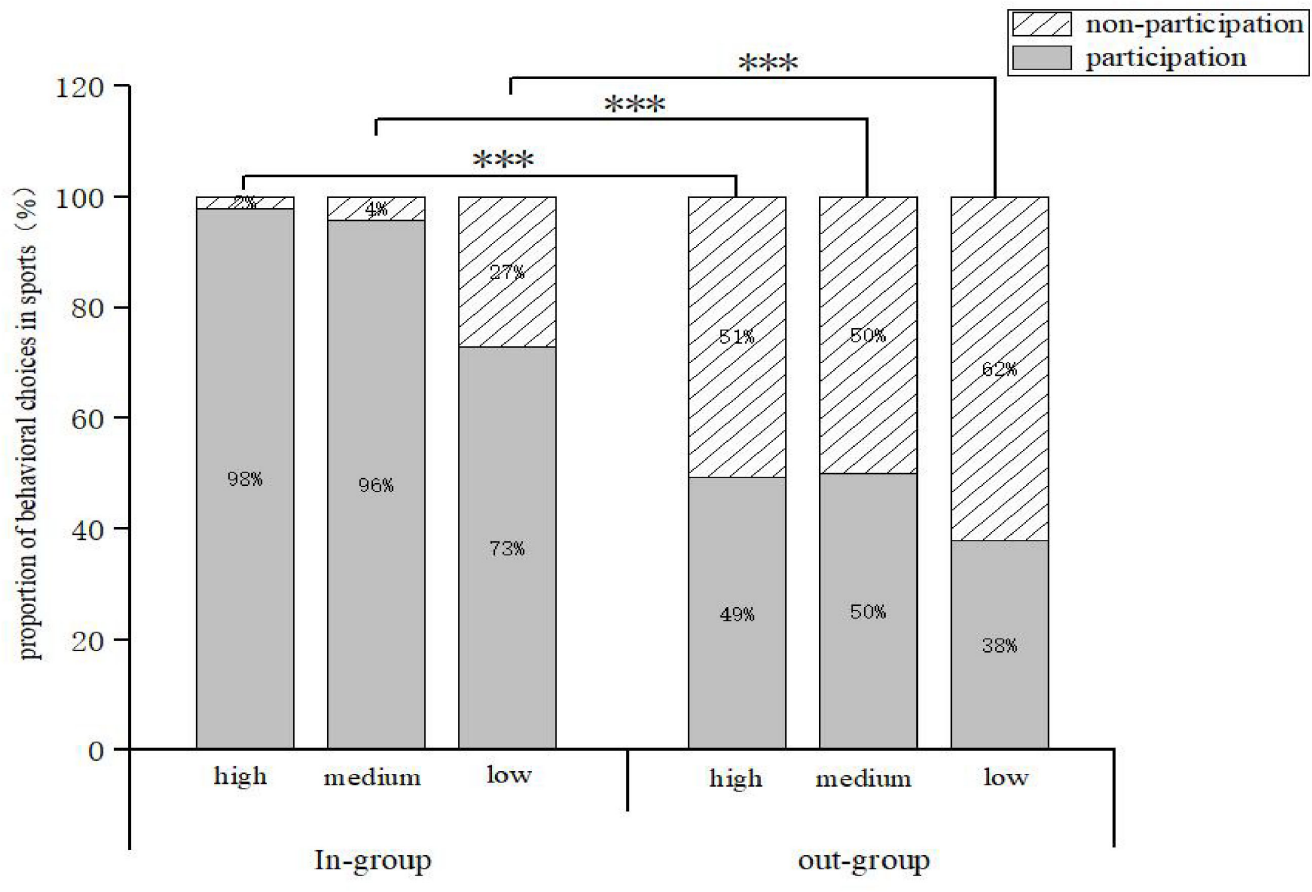
Statistical Chart of the Impact of In-Group vs. Out-Group Involvement on Behavioral Choices in Sports Programs.

A 2 (group: in-group, out-group) × 3 (sport favoritism: high favoritism, medium favoritism, low favoritism) percentage statistic was conducted for children aged 3–8 years who faced different levels of sport behavior choices in different group-involvement approach situations (Figure 4).

In the in-group, children’s participation in sports activities is high, especially in the case of high and medium favoritism, where the participation rate reaches 98% and 96%, respectively. In the out-group, children’s participation in sports activities was lower, especially in the case of low favoritism, with a participation rate of only 38%. In both the in-group and the out-group, the proportion of children’s participation in sport activities decreased accordingly with the lower degree of sport favoritism. The results showed that children’s participation was significantly higher in the in-group than in the out-group and that the level of sport enjoyment had a significant effect on participation.

A chi-square test of children’s sport item behavior under the in-group-out-group involvement approach showed that there was a significant difference between the proportion of the number of people who chose the item behavior in the in-group (98%) and the out-group (49%) with a high degree of favoritism, *Z* = –11.333b, *P* = 0.001 (Table 1). Further analysis of the number of people who itemized behavioral choices on moderate and low favorites similarly revealed a significant difference. This leads to the conclusion that children are willing to participate with the in-group in sports with a high degree of favoritism. The characteristic was also present in the medium degree of favoritism, thus validating the role of the theory of in-group favoritism. The effect of involvement through groups is different, and in-group involvement will help to enhance the positive choice of children’s sports behavior from 3 to 8 years old.

**TABLE 1:** Analysis of In-Group-Out-Group Involvement in Favoritism of Different Sports Programs.

The way of group involvement	Project preference	Negative rank	Positive rank	Fixed value	*Z*	*P*
In-groups-out-groups	High level favorite	4	140	136	−11.333^b^	0.001
	Medium favorite	6	134	140	−10.818^b^	0.001
	low favorite	8	106	166	−9.179^b^	0.001

^b^The test statistic is calculated based on positive-rank data.

### Influence of demographic variables on in-group-out-group involvement effects

A 3 (age group: 3–4 years old, 5–6 years old, 7–8 years old) × 2 (gender: male, female) × 3 (sport favoritism: high favoritism, medium favoritism, low favoritism) headcount was conducted for the selection of sports behaviors of children aged 3–8 years old by gender and age in the face of different levels of sport favoritism (Table 2), respectively. It was found that in the group condition (in-group-out-group), the results broadly showed that the in-group had a higher number of participants than the out-group in all three different favoritism levels, as well as having the phenomenon in the 3–4, 5–6, and 7–8-years-old groups at the same time. In the number of participants in the high and medium favorites, both the in-group and the out-group clearly show a higher number of participants than non-participants, and in the low favorites, both the in-group and the out-group show a significant decrease in the number of participants; there is no significant difference between the participation of boys and girls in all age groups; in general, the table shows that the in-group has a higher number of participants than the out-group in comparison with the out-group. The results in the table show that there is no difference in participation choices by gender between the in-group and the out-group.

**TABLE 2:** Analysis of children’s favoritism in different sports by gender and age.

Type of project selected	Community condition	Participation	3–4 years old (*N* = 82)		5–6 years old (*N* = 84)		7–8 years old (*N* = 114)	
			Male (*N* = 46)	Female (*N* = 36)	Male (*N* = 48)	Female (*N* = 36)	Male (*N* = 58)	Female (*N* = 56)
Highly favorite	In-groups	Participation	46	32	48	36	58	54
		non-participation	0	4	0	0	0	2
	Out-group	Participation	26	22	18	16	24	32
		non-participation	20	14	30	20	34	24
Moderate favorite	In-groups	Participation	46	32	48	36	54	52
		non-participation	0	4	0	0	4	4
	Out-group	Participation	26	22	18	18	26	30
		non-participation	20	14	30	18	32	26
Low favorite	In-groups	Participation	36	30	28	26	44	40
		non-participation	10	6	20	10	14	16
	Out-group	Participation	20	20	12	12	20	22
		non-participation	26	16	36	24	38	34

The results of Experiment 1 showed that in the in-group-out-group involvement approach, the proportion of people participating in the in-group was higher than that in the out-group in terms of the choice of sports behaviors, and there was a significant effect on the effect of group involvement in terms of the level of enjoyment of different sports; children aged 3–4 and 5–6 years old were more likely to choose to engage in sports with the in-group in terms of the choice of sports behaviors than those aged 7–8 years old, and in the case of 3–4 and 5–6-years-old children, the number of people participating in the out-group was lower, i.e., influenced by in-group preference. Children were less for out-group involvement, i.e., influenced by in-group favoritism. There are no significant age and gender differences in the choice of sports behavior in the in-group-out-group involvement approach.

Children’s choice behavior was influenced by in-group favoritism when confronted with their high, medium, and low favorites but did not show significance by age and gender, suggesting that there is no significant effect of in-group favoritism by age and gender in children’s sport behavioral choices. So, do parents and close peers in more intimate relationships show in-group favoritism among age and gender? Further examination and validation of children’s sport behavior choices is needed.

## Experiment 2

### Materials and Methods

#### Participants

Based on Study 1, the subjects were the same as those in Experiment 1, and a total of 280 children aged 3–8 years were selected from two general kindergartens and one elementary school in Nanchang City, China. 82 children were in the 3- to 4-years-old group (46 boys, mean monthly age M = 49.22, SD = 6.268); 84 children were in the 5- to 6-years-old group (48 boys, mean monthly age M = 73.10, SD = 5.28); and 114 were in the 7- to 8-years-old group (58 boys, mean monthly age M = 96.09, SD = 6.12). A 3 (age: 3–4, 5–6, 7–8) × 2 (gender: boys, girls) × 2 (group condition: parents, close peers) mixed experimental design was used, with the proportion of the number of children based on their choice of sports behavior as the outcome variable. In this case, children’s age and gender were used as between-subjects variables, and the group condition (parents, close peers) was a within-subjects variable, and all children were required to make choices for both groups. The procedure followed the Ethics Committee guidelines for human subjects.

#### Materials

The materials and procedure of the experiment were the same as in Experiment 1, and the instructions for the two stages of “differentiating the degree of enjoyment of sports” were the same as in Experiment 1. The difference between this experimental phase and Experiment 1 is the different groups oriented; children were differentiated between in-groups and out-groups in Experiment 1, whereas in this experiment the choice was made by selecting peers and parents close to the child within the scope of the in-group.

#### Procedure

Distinguish between groups (close peers vs. parents). Ask subjects to choose one close peer out of all the students in the class with whom they play the best (highest closeness). Ask the subject: “Right now, your close peer is playing your favorite (high degree of fondness) sport; would you go along to participate?” “Ask the subject: your close companion is playing your average favorite (moderate favorite) sport; would you go along to participate?” “Ask the subject: would you participate with your close companion who is playing a sport that you like the least (low favoritism)?” Test parent questioning words as above.

### Data processing

The chi-square test was used to analyze whether there was a significant difference in the proportions of children’s number of sport behavior choices in the parent and close peer involvement conditions. The Wilcoxon signed-rank test was used to compare whether the medians of the two related samples were significantly different.

## Results

### Parent-intimate peer involvement effects on behavioral choice in sports programs

A chi-square test was conducted to determine whether there was a difference in participation in the proportion of children making behavioral choices in the two group conditions (parent-intimate peer). The results show that there is no significant difference in the percentage of children aged 3–8 years between their parents (98.21%) and close peers (97.14%) participating in their high level of favorite sports, and there is no difference in the medium level of favoritism, which means that children do not have a clear preference for their parents and close peers in terms of their high and medium level of favoritism, and that children choose to participate in sports activities with both their parents and their close peers. On the low level of favoritism, children demonstrated a decreasing trend in the percentage of participants for both parents (83.57%) and close peers (74.29%), and it was also found that there was a decrease in the percentage of participation for close peers in the low level of favoritism compared to the medium level of favoritism items (Table 3).

**TABLE 3:** Analysis of parent-intimate peer involvement on behavioral choice of sports programs.

Type of project selected	Father and mother		Intimate partner	
	Frequency of participation (%)	Frequency of non-participation (%)	Frequency of participation (%)	Frequency of non-participation (%)
Highly favorite	275 (98.21)	5 (1.79)	272 (97.14)	8 (2.86)
Moderate favorite	270 (96.43)	10 (3.57)	268 (95.71)	12 (4.29)
Low favorite	234 (83.57)	46 (16.43)	208 (74.29)	72 (25.71)

A 2-sample correlation test (analysis - non-parametric test - old dialog box - 2 correlated samples) of children’s behavior in sports programs under the parent-intimate peer involvement approach showed that among the three different levels of liking, significance was shown only in the low level of liking the program only, *Z* = –2.741b, *P* = 0.006 (Table 4). Suggesting that the children’s behavior in sports programs between their parents and intimate peers in their low level of liking showed variability.

**TABLE 4:** Analysis of parent-intimate peer involvement in the selection of sports programs with different levels of liking.

The way of group involvement	Project preference	Negative rank	Positive rank	Fixed value	*Z*	*P*
Parents-close companions	Highly favorite	4	7	269	−0.905^b^	0.366
	Moderate favorite	8	10	262	−0.471^b^	0.637
	Low favorite	32	58	190	−2.741^b^	0.006

^b^The test statistic is calculated based on positive-rank data.

### The influence of demographic variables on the parent-intimate peer involvement effect

From the table averages, descriptive statistics were conducted on whether there were differences in the number of children’s sport behavior choices in the parent-intimate peer involvement condition. The results in the group condition (parents-intimate peers) showed that parents were involved in higher numbers than intimate peers in all three different levels of liking relative to intimate peers, as well as having this phenomenon simultaneously in the 3–4, 5–6, and 7–8-years-old groups. Parents and close peers showed significantly higher participation than non-participation in both high and medium favoritism; parents and close peers showed significantly lower participation in low favoritism relative to the other two levels; and there was a significant difference in age and gender only in low favoritism. More boys (42) and girls (36) in the 3–4-years-old group chose to engage with their parents than their close peers, suggesting that there is a behavioral tendency toward parental attachment in children aged 3–4 years (Table 5). Overall, the participation choices of children of different genders and ages in different levels of sport favoritism were as follows: the number of children who chose to participate with their parents was high, and there were differences in age and gender in the lower levels of favoritism.

**TABLE 5:** Behavioral analysis of children of different genders and ages in terms of their choice of different sports favorites.

Type of project selected	Community condition	Participation	3–4 years old (*N* = 82)		5–6 years old (*N* = 84)		7–8 years old (*N* = 114)	
			Male (*N* = 46)	Female (*N* = 36)	Male (*N* = 48)	Female (*N* = 36)	Male (*N* = 58)	Female (*N* = 56)
Highly Favorite	Parents	Participation	45	35	47	35	58	55
		non-participation	1	1	1	1	0	1
	Close peers	Participation	44	35	48	36	56	53
		non-participation	2	1	0	0	2	3
Moderate Favorite	Parents	Participation	46	34	46	30	58	56
		non-participation	0	2	2	6	0	0
	Close peers	Participation	46	34	46	32	56	54
		non-participation	0	2	2	4	2	2
Low favorite	Parents	Participation	42	36	38	28	44	46
		non-participation	4	0	10	8	14	10
	Close peers	Participation	36	30	36	28	42	36
		non-participation	10	6	12	8	16	20

Using the Wilcoxon signed-rank test, it was found that children’s sport behavior choices in the parent vs. close peer involvement approach showed negative, positive, and fixed values. In terms of age, it showed that a high degree of favoritism did not present a significant difference in parental and close peer involvement, X^2^ = 4.567, *P* = 0.335, indicating that in high-degree-of-favoritism sports, children are participating in activities with their parents and close peers. Age showed a significant difference on medium-degree favoritism and low-degree favoritism. In this case, on a low degree of liking, the negative rank is less than the positive rank in 3–4- and 5–6-years-olds, which indicates that we find a tendency of attachment behavior toward parents in younger children, which decreases with increasing age. In terms of age, children did not show significance between parents and close peers in terms of gender (Table 6).

**TABLE 6:** Differential analysis of parent-intimate peer involvement on behavioral choices of sports events.

The way of group involvement	Type of project selected	Index	Age			Gender	
			3–4 years old (*N* = 82)	5–6 years old (*N* = 84)	7–8 years old (*N* = 114)	Male (*N* = 152)	Female (*N* = 128)
Parents-close companions	Highly Favorite	Negative rank	1	2	1	1	3
		Positive rank	2	0	5	3	4
		Fixed value	79	82	108	148	121
		*X*^2^/*P*	4.567/0.335			1.809/0.405	
	Moderate Favorite	Negative rank	2	6	0	2	6
		Positive rank	2	4	4	4	6
		Fixed value	78	74	110	146	116
		*X*^2^/*P*	9.730/0.045			3.806/0.149	
	Low favorite	Negative rank	2	18	12	20	12
		Positive rank	14	20	24	30	28
		Fixed value	66	46	78	102	88
		*X*^2^/*P*	18.205/0.001			1.051/0.591	

Experiment 2 explored children’s sport behavior choices under the parent-intimate peer involvement approach. The results showed that children’s sport behavior choices under the parent-peer involvement approach were not significant in the high degree of favoritism and medium degree of favoritism items in age and gender, and there was a significant difference in sport behavior choices by age only in the low degree of favoritism items. Moreover, the number of parental involvement was higher than the number of close peer involvement in the low degree of favoritism program, and this characteristic was especially preferred with the involvement of parents in the age groups of 3–4- and 6–7 years old, which demonstrated that the children were attached.

## Discussion

### The effect of different group involvement on the behavioral choice of sports programs

This study examined the influence of group preferences on children’s sports behavior choices through two types of participation, namely, in-group and out-group participation patterns. Specifically, it investigated whether children’s sports behavior choices are influenced by groups when faced with different group participation patterns. The results of this study indicate that group preferences can form as early as 3–4 years of age through simple group categorization, without the need for direct intergroup contact. This finding contradicts traditional contact theory (Pettigrew, 1998). Contact theory posits that meaningful social identification may form prior to physical contact between groups and can develop independently of physical contact between groups. At all preference levels, the significant differences between ingroup and outgroup participation strongly support social identity theory and self-classification theory (Hornsey, 2008), particularly their predictions regarding the primacy of group identity in shaping behavior. The findings of this study are consistent with recent research in developmental psychology, which suggests that even arbitrary group distinctions can trigger young children’s preference for ingroups (Thomas, 2024), indicating that these processes form the foundation of early social cognition. Children consistently tend to engage in physical activities with ingroup members, with participation rates in highly preferred activities reaching 98% within the ingroup, compared to 49% in the outgroup. This pattern holds across all age groups, although the intensity decreases in older children, which may reflect the developmental complexity of group evaluation cognition (Clark and Delia, 1977). The study also found that in-group preference is reflected in different sports behavior choices, such as social skills and sharing (Yu et al., 2016; Corbit et al., 2022). Additionally, in group settings, individuals tend to express dislike toward outgroup members in order to maintain their positive image or status within the group. Research indicates that 3- and 4-years-old children are more likely to share their favorite items with ingroup members while sharing disliked items with outgroup members (Buttelmann and Böhm, 2014). Compared to ingroups, perceived advantages or disadvantages of outgroups can trigger different negative emotions, such as anger, disgust, or fear (Mackie et al., 2000). The above research indicates that the children in this study exhibited more active participation in physical activities among members of their ingroup. These findings suggest that there is a strong ingroup preference in early childhood, the expression of which is inevitably filtered through the lens of China’s collectivist culture. The emphasis on classroom unity in Chinese education may particularly reinforce ingroup preferences relative to cultures that value individual achievement.

Experiment 2 examined children’s sport behavior choices under a parent-intimate peer involvement approach. It was found that there was no significant difference between high degree of favoritism and medium degree of favoritism in sports, which on the other hand shows that relative to the in-group-out-group involvement approach, the parent-intimate-peer closeness relationship is higher to the extent that there is no significant difference in sports. Significant differences were presented only on the low degree of favorite sports. It has been established that children exhibit different motivations and goals when confronted with their favorite sports (Schorno et al., 2022). Children are motivated to participate in physical activity for a variety of reasons, not least to have fun. Previous research has found that having fun is often cited as the main reason children begin and subsequently maintain sport participation (Visek et al., 2015). Conversely, when there is a lack of fun, children tend to lose interest and drop out of physical activity or engage in other activities that they find more enjoyable (Crane and Temple, 2015). It was concluded that children were not as interested in the low level of favorite sports as they were in the high level of favorites, and that there was a corresponding decrease in the number of children choosing to participate when faced with a low level of favorites, which may be related to their interests. In summary, the higher level of sports behavior choices and less group influence in older children may be explained by the fact that younger children are still developing cognitively and their psycho-theoretical abilities are still to be improved.

### Influence of demographic variables on group involvement effects

This study verified that children’s sport behavioral choices based on group preference theory exhibited age differences through two different group involvement situations. The present study found that children in the 7–8-years-old group were found to be less affected by the degree of in-group favoritism in the in-group versus out-group involvement approach than children in the 3–4 and 5–6-years-old groups. This developmental pattern may reflect several interconnected processes: (1) advancing social-cognitive abilities that allow older children to consider multiple social categories simultaneously (e.g., group membership alongside individual preferences); (2) the expansion of peer networks in middle childhood that dilutes strong in-group attachments; and (3) emerging critical thinking skills that enable more autonomous decision-making independent of group influence. Children’s sport behavior choices have been linked to group involvement effects. Research has found that individual preferences for groups are more expected to do the same things as the group and treat things within the group more positively (Killen et al., 2013). Previous research has also found that children become friends with children who share both favorite foods and toys at the age of 3 (Fawcett and Markson, 2010), and conversely, children who play sports with their favorite friends (in-groups) show more positive behavior. There are studies that also prove the point of view that children prefer to associate their positive behavior with in-groups and not with out-groups relative to them (Dunham et al., 2011). Identity and shared preferences of social group members influence children’s behavior (Sparks et al., 2017). The effect of in-group favoritism changes with age, with in-group favoritism increasing with age in girls and decreasing with age in boys (Misch et al., 2022). This is partially similar to the findings in the research section of this paper, where the effect of in-group favoritism becomes smaller the older the child. The reason for this age trend may be that, with increasing age, children’s choices of sports programs and groups become more rational, i.e., children show their own perceptions of having their own insights into the way they play sports and their choice of sports behaviors. Cognitively and emotionally, children’s in-group favoritism is presented as showing stronger in-group favoritism at the age of 6 than at the age of 3, possibly due to a shift from the individual level to the group level during childhood (Dunham and Emory, 2014), a view that supports this paper. The results between parent-intimate peers in Experiment 2 showed that children aged 3–4 and 5–6 years chose to engage in sport behaviors with their parents more than children aged 7–8 years, indicating that children at younger ages were more inclined to engage in participatory sport behaviors with their parents. Because children’s learning and living environments change significantly after age 7, they spend more time communicating and interacting with their peers, so they are more emotionally attached to their peers after age 7. The parent-peer shift observed in Experiment 2 (from parental to peer attachment between ages 3–6 and 7–8) may reflect an interaction between biological maturation (e.g., adrenarche) and social ecology (e.g., school transitions), suggesting future research should examine biopsychosocial models of group preference development (Haslam et al., 2019).

### The effects of parental attachment and peer attachment on children’s sport behavior choices

The results of Experiment 2 showed that children in the 3–4 and 5–6 age groups were more inclined to participate in sports behaviors with their parents, while children in the 7–8 age group were more inclined to go with their peers to participate in sports behaviors. This may be closely linked to attachment. Attachment theory emphasizes the impact of attachment relationships on an individual’s personality and social understanding, with the idea that individuals already seek closeness between attachment figures from birth (Wang, 2019). Parents and peers play a vital role in influencing the development of an individual. Relevant studies have pointed out that the quality of parent-child attachment affects a person’s social adaptability (Liang, 2017). At the same time, peer relationships affect the development of social adaptability and academic achievement of elementary school students (Zhang, 2008). Peer attachment is an emotional relationship with peers that gradually develops during an individual’s growth process as feelings for parents are extended to interactions with peers (Xiang et al., 2021). In the study, it was found that children aged 7–8 years old have some connection with their peers, and their mutual connection is lasting and stable, which includes trust, dependence, and sharing of personal feelings and thoughts, which is consistent with the viewpoints of research scholars (Zhong et al., 2014) and others. To summarize, having good parent-child and peer relationships helps individuals to build better interpersonal relationships.

In the midst of the experiment, this study found that children’s interactions with their close peers come partly from trust and security in their peers, and that this environment promotes children’s positive interaction behaviors, which leads to sports behavior participation. In the results of the experiment it was found that children’s attachment to their parents was mainly concentrated in the lower ages, and in the higher ages children’s social relationships became enriched with the gradual formation of relationships with classmates, friends, and peers.

### Limitations

First, the study sample was limited to 280 children aged 3–8 in two general kindergartens and one elementary school in Nanchang. The limited geographic scope of the sample may not be representative of other regions or groups of children in different cultural contexts. Future research should expand this work through cross-cultural comparisons (e.g., Western vs. Eastern contexts), inclusion of rural populations, and examination of how varying school environments and physical education policies might moderate these effects.

Second, this study focuses on two experimental situations (in-group-out-group involvement approach and parent-intimate peer involvement approach) to explore the effects of different group involvement on children’s sport behavior choices. Although these two contexts can reveal some group favoritism phenomena, real-life group involvement contexts are far more complex and diverse than the experimental settings. Future studies should employ naturalistic observation and experience sampling methods to examine how multiple, overlapping group memberships influence children’s sport choices in real-world settings.

Finally, the measurements of children’s sport behavioral choices in the study relied primarily on choice behavior and willingness to participate during the experiment, without delving into the psychological mechanisms, cognitive processing, and affective experiences inherent in children’s choices. Future research should combine behavioral observations with interviews and physiological measures to better understand children’s motivational and emotional processes in sports choices.

## Conclusion

1. Of the in-group and out-group involvement approaches, in-group involvement will help to enhance the positive selection of and participation in physical education behaviors of 3- to 8-years-old children.

2. Children’s sport behavior choices are influenced by group favoritism, an influence that manifests itself primarily in terms of in-groups, parents, and close peers.

3. Among the parent-intimate peer involvement modalities, 3- to 4- and 5- to 6-year-old children exhibit parent-attachment behaviors and may develop peer attachment with age.

## Data Availability

The original contributions presented in this study are included in this article/supplementary material, further inquiries can be directed to the corresponding author.
